# Supplementary material for: Heparin-based hydrogel scaffolding alters the transcriptomic profile and increases the chemoresistance of MDA-MB-231 triple-negative breast cancer cells
Source: Biomater Sci. 2020 Feb 13;8(10):2786–96. doi: 10.1039/c9bm01481k (PMC7497406; doi:10.1039/c9bm01481k)
Supplement: Supplementary file 2 [file BM-008-C9BM01481K-s002.zip › Supplementary File 4/HP-B hydrogel + EGF (s)vControl/Pathways/my_analysis.Gsea.1545201170987/HALLMARK_SPERMATOGENESIS.html]

Details for gene set HALLMARK\_SPERMATOGENESIS[GSEA]

|  || Dataset | expr.class.cls#HYDEGF\_versus\_CONTROL.class.cls#HYDEGF\_versus\_CONTROL\_repos |
| Phenotype | class.cls#HYDEGF\_versus\_CONTROL\_repos |
| Upregulated in class | HYDEGF |
| GeneSet | HALLMARK\_SPERMATOGENESIS |
| Enrichment Score (ES) | 0.25537512 |
| Normalized Enrichment Score (NES) | 1.0048879 |
| Nominal p-value | 0.4168126 |
| FDR q-value | 0.5682124 |
| FWER p-Value | 1.0 |
Table: GSEA Results Summary

  

Fig 1: Enrichment plot: HALLMARK\_SPERMATOGENESIS      
 Profile of the Running ES Score & Positions of GeneSet Members on the Rank Ordered List

  

| PROBE | DESCRIPTION (from dataset) | GENE SYMBOL | GENE\_TITLE | RANK IN GENE LIST | RANK METRIC SCORE | RUNNING ES | CORE ENRICHMENT || 1 | CHFR | na |  |  | 578 | 2.037 | 0.0094 | Yes |
| 2 | PGS1 | na |  |  | 848 | 1.783 | 0.0301 | Yes |
| 3 | SCG5 | na |  |  | 904 | 1.747 | 0.0612 | Yes |
| 4 | PHKG2 | na |  |  | 1033 | 1.665 | 0.0870 | Yes |
| 5 | MTOR | na |  |  | 1080 | 1.635 | 0.1164 | Yes |
| 6 | NF2 | na |  |  | 1227 | 1.555 | 0.1391 | Yes |
| 7 | EZH2 | na |  |  | 1241 | 1.545 | 0.1685 | Yes |
| 8 | MAST2 | na |  |  | 1359 | 1.488 | 0.1914 | Yes |
| 9 | CCNA1 | na |  |  | 1442 | 1.452 | 0.2154 | Yes |
| 10 | DDX25 | na |  |  | 1577 | 1.401 | 0.2356 | Yes |
| 11 | CNIH2 | na |  |  | 1981 | 1.273 | 0.2394 | Yes |
| 12 | ACRV1 | na |  |  | 2360 | 1.162 | 0.2422 | Yes |
| 13 | GFI1 | na |  |  | 2553 | 1.113 | 0.2539 | Yes |
| 14 | CLPB | na |  |  | 3373 | 0.927 | 0.2291 | Yes |
| 15 | PHF7 | na |  |  | 3521 | 0.901 | 0.2390 | Yes |
| 16 | GAD1 | na |  |  | 3542 | 0.896 | 0.2554 | Yes |
| 17 | SLC12A2 | na |  |  | 3959 | 0.820 | 0.2496 | No |
| 18 | BUB1 | na |  |  | 4319 | 0.763 | 0.2457 | No |
| 19 | JAM3 | na |  |  | 5275 | 0.610 | 0.2076 | No |
| 20 | PAPOLB | na |  |  | 5360 | 0.596 | 0.2148 | No |
| 21 | HSPA4L | na |  |  | 5521 | 0.573 | 0.2176 | No |
| 22 | KIF2C | na |  |  | 5742 | 0.538 | 0.2166 | No |
| 23 | STRBP | na |  |  | 6142 | 0.483 | 0.2052 | No |
| 24 | DBF4 | na |  |  | 6300 | 0.463 | 0.2060 | No |
| 25 | PIAS2 | na |  |  | 6679 | 0.413 | 0.1943 | No |
| 26 | NCAPH | na |  |  | 6909 | 0.384 | 0.1898 | No |
| 27 | HSPA2 | na |  |  | 7157 | 0.351 | 0.1837 | No |
| 28 | SNAP91 | na |  |  | 7332 | 0.324 | 0.1809 | No |
| 29 | TOPBP1 | na |  |  | 7340 | 0.323 | 0.1868 | No |
| 30 | MLLT10 | na |  |  | 7386 | 0.319 | 0.1907 | No |
| 31 | ZC3H14 | na |  |  | 7941 | 0.250 | 0.1666 | No |
| 32 | OAZ3 | na |  |  | 8395 | 0.199 | 0.1468 | No |
| 33 | GPR182 | na |  |  | 8444 | 0.196 | 0.1481 | No |
| 34 | PCSK4 | na |  |  | 8480 | 0.191 | 0.1500 | No |
| 35 | HSPA1L | na |  |  | 8730 | 0.160 | 0.1401 | No |
| 36 | CAMK4 | na |  |  | 9137 | 0.111 | 0.1210 | No |
| 37 | MLF1 | na |  |  | 9226 | 0.100 | 0.1183 | No |
| 38 | LPIN1 | na |  |  | 9566 | 0.067 | 0.1019 | No |
| 39 | PRKAR2A | na |  |  | 9800 | 0.035 | 0.0904 | No |
| 40 | AGFG1 | na |  |  | 9825 | 0.031 | 0.0898 | No |
| 41 | COIL | na |  |  | 10358 | -0.029 | 0.0625 | No |
| 42 | MAP7 | na |  |  | 10826 | -0.082 | 0.0397 | No |
| 43 | CDKN3 | na |  |  | 10895 | -0.093 | 0.0379 | No |
| 44 | CCNB2 | na |  |  | 11220 | -0.126 | 0.0234 | No |
| 45 | PSMG1 | na |  |  | 11242 | -0.129 | 0.0249 | No |
| 46 | AURKA | na |  |  | 11282 | -0.134 | 0.0254 | No |
| 47 | SEPT4 | na |  |  | 11397 | -0.146 | 0.0223 | No |
| 48 | GMCL1 | na |  |  | 11425 | -0.149 | 0.0238 | No |
| 49 | PARP2 | na |  |  | 11606 | -0.176 | 0.0178 | No |
| 50 | CSNK2A2 | na |  |  | 11652 | -0.181 | 0.0190 | No |
| 51 | IP6K1 | na |  |  | 11834 | -0.208 | 0.0136 | No |
| 52 | BRAF | na |  |  | 12466 | -0.281 | -0.0139 | No |
| 53 | SIRT1 | na |  |  | 12672 | -0.309 | -0.0186 | No |
| 54 | NEFH | na |  |  | 13072 | -0.352 | -0.0326 | No |
| 55 | RAD17 | na |  |  | 13096 | -0.353 | -0.0270 | No |
| 56 | ARL4A | na |  |  | 13134 | -0.358 | -0.0219 | No |
| 57 | TTK | na |  |  | 13159 | -0.362 | -0.0161 | No |
| 58 | NEK2 | na |  |  | 13399 | -0.403 | -0.0208 | No |
| 59 | TCP11 | na |  |  | 13583 | -0.423 | -0.0221 | No |
| 60 | RFC4 | na |  |  | 13625 | -0.429 | -0.0159 | No |
| 61 | PCSK1N | na |  |  | 13873 | -0.470 | -0.0197 | No |
| 62 | VDAC3 | na |  |  | 14280 | -0.516 | -0.0308 | No |
| 63 | IFT88 | na |  |  | 14858 | -0.613 | -0.0491 | No |
| 64 | TLE4 | na |  |  | 14968 | -0.629 | -0.0425 | No |
| 65 | IDE | na |  |  | 15687 | -0.759 | -0.0653 | No |
| 66 | CDK1 | na |  |  | 15956 | -0.811 | -0.0635 | No |
| 67 | TALDO1 | na |  |  | 16127 | -0.852 | -0.0558 | No |
| 68 | RPL39L | na |  |  | 16273 | -0.892 | -0.0460 | No |
| 69 | TSN | na |  |  | 16652 | -0.987 | -0.0465 | No |
| 70 | GSTM3 | na |  |  | 16738 | -1.008 | -0.0314 | No |
| 71 | SLC2A5 | na |  |  | 17113 | -1.116 | -0.0292 | No |
| 72 | CCT6B | na |  |  | 17160 | -1.131 | -0.0096 | No |
| 73 | CLGN | na |  |  | 17250 | -1.161 | 0.0084 | No |
| 74 | STAM2 | na |  |  | 17437 | -1.229 | 0.0226 | No |
| 75 | PEBP1 | na |  |  | 17889 | -1.434 | 0.0269 | No |
| 76 | NPHP1 | na |  |  | 18761 | -2.128 | 0.0229 | No |
Table: GSEA details [plain text format]

  

Fig 2: HALLMARK\_SPERMATOGENESIS      
 Blue-Pink O' Gram in the Space of the Analyzed GeneSet

  

Fig 3: HALLMARK\_SPERMATOGENESIS: Random ES distribution      
 Gene set null distribution of ES for **HALLMARK\_SPERMATOGENESIS**
